# Histone modifications centric-regulation in osteogenic differentiation

**DOI:** 10.1038/s41420-021-00472-6

**Published:** 2021-05-03

**Authors:** Kun Li, Jinxiang Han, Ziqiang Wang

**Affiliations:** 1 Department of Nuclear Medicine, The First Affiliated Hospital of Shandong First Medical University & Shandong Provincial Qianfoshan Hospital, 250014 Jinan, China; 2 Biomedical Sciences College & Shandong Medicinal Biotechnology Centre, Shandong First Medical University & Shandong Academy of Medical Sciences, 250062 Jinan, China

**Keywords:** Histone post-translational modifications, Differentiation

## Abstract

Histone modification critically contributes to the epigenetic control of gene expression by changing the configuration of chromatin and modifying the access of transcription factors to gene promoters. Recently, we observed that histone acetylation and crotonylation mediated the expression of endocytosis-related genes and tumor-related immune checkpoint genes by regulating the enrichment of signal transducer and activator of transcription 3 on these gene promoters in Alzheimer’s disease and tumorigenesis, suggesting that histone modification plays an important role in disease development. Furthermore, studies performed in the past decade revealed that histone modifications affect osteogenic differentiation by regulating the expression of osteogenic marker genes. In this review, we summarize and discuss the histone modification-centric regulation of osteogenic gene expression. This review improves the understanding of the role of histone modifications in osteogenic differentiation and describes its potential as a therapeutic target for osteogenic differentiation-related diseases.

## Facts

- The levels of histone modifiers and their regulators are altered during osteogenic differentiation and the development of osteogenic differentiation-related diseases.
- Histone modifications orchestrate osteogenic differentiation.
- Histone modifications regulate the expression of osteogenic marker genes by affecting the chromatin structure and transcription factor activity.

## Open questions

- Which types of histone modifications are mainly responsible for osteogenic differentiation?
- How do histone modifications coordinate to regulate the expression of osteogenic marker genes?
- Can histone modifications be targeted for treating osteogenic differentiation-related diseases?

## Introduction

Histone modification is an important epigenetic process with a key role in diverse biological processes, including transcription, chromosome packaging, and DNA repair^1–3^. Histone acetylation and methylation are the most widespread and dynamic histone modifications. Histone acetyltransferases and histone deacetylases mediate the addition and removal of acetyl groups at lysine residues in the N-terminal tails of histones, respectively, and histone methyltransferase and histone demethylase mediate the addition and removal of methyl groups at lysine and arginine residues, respectively^4,5^. Increasing evidence has demonstrated that histone modification is involved in multiple pathological processes, including viral infection, inflammation, neurodegenerative diseases, and tumorigenesis^6–10^. Our previous study of epigenetic regulation in Alzheimer’s disease and T cell exhaustion showed that histone acetylation is involved in long non-coding RNA NEAT1- and splicing factor SRSF2-mediated expression of endocytosis-related genes and tumor-related immune checkpoint genes, respectively, by recruiting signal transducer and activator of transcription 3 to these gene promoters^11,12^.

Osteogenic differentiation is a key process in bone formation, and its dysfunction leads to bone metabolism-related diseases^13–15^. During osteogenic differentiation, some transcription factors and signaling pathways are required for the expression of osteogenic genes, such as runt-related transcription factor 2 (Runx2), bone morphogenetic protein (BMP) signaling, and Wnt signaling^16–18^. Runx2 is a mammalian homolog of the *Drosophila* runt^19^, which functions as a transcription factor required to initiate osteogenic differentiation by regulating the expression of osteogenic marker genes^20–23^. BMP signaling is a central pathway that induces osteogenic differentiation and bone formation^24–27^, partly by enhancing the expression of Runx2 by stimulating p300-mediated Runx2 acetylation, inhibiting Smurf1-mediated degradation of Runx2, and promoting the transcription of Runx2^28^. Wnt signaling is another important pathway for osteogenic differentiation and bone formation. The canonical Wnt pathway can activate the expression of Runx2 to promote osteogenic differentiation of bone mesenchymal stem cells (BMSCs)^29,30^. An increasing number of studies have shown that histone modification is a potential mediator of these regulations by affecting chromatin structure and transcription factor activity^31–33^.

In this review, we summarize and discuss the regulators and histone modifiers that are altered with modulated levels of histone modification observed during osteogenic differentiation and osteogenic differentiation-related diseases. Additionally, we also discuss the molecular mechanisms by which the regulated histone modification mediates the expression of osteogenic marker genes that affect osteogenic differentiation.

### Histone acetylation

Histone acetylation modification, a key mechanism of epigenetic regulation, is closely related to the activation of osteogenic marker gene expression during osteogenic differentiation and bone formation^34–36^. Furthermore, specific sites of histone acetylation, such as H3K9Ac, have been reported to be involved in this process. In this section, we summarize and discuss the regulators and histone modifiers that modulate H3K9Ac, and their roles in the expression of osteogenic marker genes (Table 1).

**Table 1:** Histone acetylation centric-regulation in osteogenic genes expression.

Regulator/histone modifer	Histone modification	Target gene	Role	Reference
PCAF	H3K9Ac	BMP2, BMP4,BMPR1B, Runx2	Promoted osteogenic differentiation of BMSCs and bone formation	^38^
GCN5		Wnt1, Wnt6, Wnt10a, Wnt10b	Promoted osteogenic differentiation of BMSCs	^43^
HDAC1		Runx2, OSX, OC, P27	Inhibited osteogenic differentiation of BMSCs	^44^
NAMPT		Runx2	Promoted osteogenic differentiation of BMSCs	^47^
MAPK		BGLAP2, IBSP	Promoted osteogenic differentiation of preosteoblast cells	^53^
MAPK	H4K5Ac	Bglap2, LBSP	Promoted osteogenic differentiation of preosteoblast cells	^53^

H3K9Ac is a hallmark of gene activation and is highly correlated with active promoters^37^. To date, several regulators- and histone modifiers-mediated H3K9Ac, have been reported to be involved in the regulation of osteogenic marker gene expression during osteogenic differentiation. A study, aimed at understanding the molecular mechanism of epigenetic regulation in the osteogenic differentiation of mesenchymal stem cells (MSCs), showed that the histone acetyltransferase PCAF promoted the expression of BMP pathway genes, including *BMP2*, *BMP4*, *BMPR1B*, and *Runx2*, by increasing histone H3K9 acetylation in the promoter regions of these genes^38^. In osteoporosis, a common degenerative bone disease characterized by disrupted osteogenesis and resorption^39–41^, the expression of GCN5, another histone acetyltransferase that is 73% homologous to PCAF^42^, was reportedly decreased. This decrease inhibited the osteogenic differentiation of BMSCs by reducing the acetylation of H3K9 on the promoters of Wnt genes such as *Wnt1, Wnt6, Wnt10a*, and *Wnt10b*^43^.

In addition, a study investigating the role of modifications in the chromatin structure in osteogenic differentiation revealed that the expression of histone deacetylase 1 and its enzymatic activity were significantly decreased, resulting in induction of the osteoblast marker genes *Runx2*, osterix (*OSX*), and osteocalcin (*OC*), and cell cycle arrest gene *P27*, by enhancing H3K9Ac at these gene promoters^44^. Nicotinamide phosphoribosyltransferase, an enzyme involved in nicotinamide adenine dinucleotide biosynthesis^45^, was found to be upregulated during the osteogenic differentiation of multi- and omnipotent progenitors^46^. Investigations of the molecular mechanism revealed that nicotinamide phosphoribosyltransferase promoted osteogenic differentiation by increasing the enrichment of H3K9Ac at the Runx2 promoter, thus enhancing Runx2 transcriptional activity^47^. The mitogen-activated protein kinase pathway mediates osteogenic differentiation^48^. Further mechanistic studies revealed that mitogen-activated protein kinase enhances the expression of osteoblast marker genes, such as bone gamma-carboxyglutamate protein 2 and integrin-binding sialoprotein, by elevating H3K9Ac and H4K5Ac, another hallmark of gene activation^49,50^, which is catalyzed by Tip60 and CBP/p300^51,52^ at these gene promoters and enhancers by associating with Runx2, as well as by mediating Runx2 S301/S319 phosphorylation^53–55^.

#### Histone methylation

Histone methylation is another histone modification involved in regulating osteogenic marker gene expression. It is closely related to the activation or suppression of osteogenic marker gene expression. Methylation of H3K9, H3K27, and H4K20 is often associated with inactive chromatin, whereas methylation of H3K4, H3K36, H3K79, and H3R17 is largely associated with active gene transcription^56^. In this section, we summarize the regulators and histone modifiers that modulate H3K4, H3K9, and H3K27 methylation, and their roles in the expression of osteogenic marker genes (Table 2).

**Table 2:** Histone methylation centric-regulation in osteogenic genes expression.

Regulator/histone modifer	Histone modification	Target gene	Role	Reference
RBP2	H3K4me3	OC, OSX, Runx2	Inhibited osteogenic differentiation of hASCs	^58,59^
BCOR		AP-2, EREG	Decreased osteo/dentinogenic potentials of MSCs	^62,63^
Ash1l		OSX, Runx2, HOXA10, Sox9	Promoted osteogenic differentiation	^65^
NO66		BSP, OC	Inhibited osteogenic differentiation of osteoprogenitor cells and mineralization	^66^
KDM7A	H3K9me2	C/ebpα, SFRP1	Inhibited osteogenic differentiation of osteoprogenitor cells	^73^
KDM4B	H3K9me3	DLX5	Promoted osteogenic differentiation of MSCs	^69^
KDM4A		C/ebpα, SFRP4	Inhibited osteogenic differentiation of osteoprogenitor cells	^74^
KDM7A	H3K27me2	C/ebpα, SFRP1	Inhibited osteogenic differentiation of osteoprogenitor cells	^73^
KDM6B	H3K27me3	HOXC6-1	Promoted osteogenic differentiation of MSCs	^69^
CDK1		HOXA7, HOXA9,Runx2, TCF7	Promoted osteogenic differentiation of MSCs	^79^
EZH2		Runx2, OC, ZBTB16, MX1, FHL1	Inhibited osteogenic differentiation of MSCs	^76–78^
KDM6A		Runx2, OC	Promoted osteogenic differentiation of MSCs	^76^
TNFα		Runx2	Inhibited osteogenic differentiation of rMSCs	^80^
DLX3		DKK4	Promoted osteogenic differentiation of BMSCs	^82^
BCOR	H3K36me2	AP-2, EREG	Inhibited osteogenic differentiation of MSCs	^62,63^
NO66	H3K36me3	BSP, OC	Inhibited osteogenic differentiation of preosteoblasts	^66^

#### H3K4 methylation

The first modulator of H3K4 methylation that participates in osteogenic differentiation is retinoblastoma binding protein 2 (RBP2), also known as lysine (K)-specific demethylase 5A, which specifically catalyzes the demethylation of dimethyl or trimethyl histone H3 lysine 4 (H3K4me2 or H3K4me3)^57^. In a study examining the epigenetic regulation of osteogenic differentiation of human adipose-derived stromal cells, RBP2 was found to negatively regulate the transcription of osteogenic marker genes, *OC* and *OSX*, by physically associating with their gene promoters to reduce the level of H3K4me3^58^. In another study of the underlying molecular mechanisms of osteoporosis, RBP2 was shown to be upregulated during osteoporosis. This resulted in inhibition of BMP2-induced osteogenic differentiation. A molecular mechanism study demonstrated that RBP2 repressed osteogenic differentiation by decreasing H3K4me3 levels on the promoters of Runx2, thus inhibiting Runx2 transcription^59^.

The BCL6 co-repressor (BCOR) represses gene transcription by associating with the transcription repressor BCL-6^60,61^. A study of the roles of BCOR mutations in oculo-facio-cardio-dental syndrome, a rare genetic disorder characterized by canine teeth with extremely long roots, and eye, craniofacial, and cardiac abnormalities, revealed that the BCOR mutation enhanced the osteogenic capacity of MSCs by promoting the expression of AP-2α, a key factor that mediates the osteo/dentinogenic differentiation of MSCs by interfering with the interaction of FBXL10, also known as Jumonji C histone demethylase 1B with the AP-2α promoter, thereby increasing H3K4me3 and H3K36me2 levels at this promoter^62^. Another study revealed that BCOR associates with FBXL11, a paralog of FBXL10, also known as histone demethylase, K-specific demethylase 2A, to inhibit transcription of the epidermal growth factor EREG by decreasing H3K4me3 and H3K36me2 levels at the EREG promoter. This change results in inhibition of the osteogenic differentiation potential of MSCs^63^.

Additionally, absent, small, or homeotic disc1-like (Ash1l), a member of the Trx family, was found to promote gene expression via the methyltransferase activity of its SET domain^64^. In a study investigating the role of Ash1l in the differentiation of MSCs, Ash1l enhanced the osteogenic and chondrogenic differentiation of C3H10T1/2 cells by increasing the enrichment of H3K4me3 at the osteogenic marker gene promoters, including OSX, Runx2, Hoxa10, and Sox9^65^. OSX is an important transcription factor required for osteogenic differentiation and bone formation. In a study performed to understand the regulatory roles of OSX in osteogenic differentiation, the JmjC domain-containing protein NO66 was found to function as a histone demethylase, with reported involvement in osteogenic differentiation and maturation of preosteoblasts by interacting with OSX and regulating OSX target genes, including BSP and OC, by modulating H3K4me3 and H3K36me3 levels at these gene promoters^66^.

#### H3K9 methylation

In BMP-stimulated osteogenic differentiation of MSCs, histone K-specific demethylase 4B (KDM4B), also known as JMJD2B, and histone K-specific demethylase 6B (KDM6B), also known as JMJD3, were found to be upregulated and essential for the osteogenic differentiation of MSCs and bone formation. Mechanistically, KDM4B enhanced the expression of DLX5, which mediates osteogenic differentiation in an OSX-dependent manner^67,68^ by removing H3K9me3 from the gene promoter, whereas KDM6B enhanced the expression of HOXC6-1, a homeodomain-containing transcription factor playing a critical role in osteogenic differentiation by removing H3K27me3 from the gene promoter^69^.

Furthermore, Wang et al. observed that histone K-specific demethylase 7A (KDM7A) binds to the promoter of CCAAT/enhancer-binding protein α (C/EBPα) and secretes frizzled-related protein 1, which both attenuate the canonical Wnt signaling pathway and play important roles in both adipogenesis and osteogenesis^70–72^, to promote gene transcription by removing the histone methylation marks H3K9me2 and H3K27me2 at the gene promoter^73^. They also found that histone K-specific demethylase 4A, also known as JMJD2A, functions to repress Wnt signaling, thereby blocking osteogenic differentiation via enhancing the transcription of C/EBPα and secreted frizzled-related protein 4 by removing H3K9me3 at the gene promoter^74^.

#### H3K27 methylation

Enhancer of Zeste homology 1 and 2 (EZH1 and EZH2) are methyltransferases that methylate histone 3 lysine 27 on chromatin to repress target gene expression^75^. To date, several studies have reported that EZH2 is involved in osteogenic differentiation through the epigenetic regulation of osteogenic marker genes via its methyltransferase activity. They found that EZH2 inhibits expression of the osteogenic marker genes Runx2, OC, ZBTB16, MX1, and FHL1 to inhibit osteogenic differentiation by increasing H3K27me3 levels at these gene promoters^76–78^, whereas lysine demethylase 6A induces osteogenic differentiation by removing this repressive mark from Runx2 at the OC gene promoter^76^. Moreover, cyclin-dependent kinase 1 has been found to enhance MSC differentiation into osteoblasts in an EZH2-dependent manner. It was observed that cyclin-dependent kinase 1 inhibited EZH2 methyltransferase activity by promoting the phosphorylation of EZH2 at Thr 487, thus disrupting EZH2 binding with other components of polycomb repressive complex 2. This resulted in upregulation of the osteogenic marker genes *HOXA7, HOXA9, Runx2*, and *TCF7* because of reduction in H3K27Me3 levels at these gene promoters^79^.

In addition, a study of rat BMSCs revealed that tumor necrosis factor-α inhibited osteogenic differentiation by increasing enrichment of H3K27me3 at the Runx2 gene promoter, thus attenuating Runx2 gene expression^80^. In another study to clarify the molecular mechanism through which distal-less homeobox 3 (DLX3), a DLX family transcription factor, modulates the osteogenic differentiation of BMSCs, DLX3 was found to promote the osteogenic differentiation of BMSCs by inhibiting the expression of Dickkopf 4, an antagonist of the Wnt/β-catenin pathway by disrupting the interaction of Wnt ligands with LRP5/6^81^, via increasing the enrichment of H3K27me3 at the Dickkopf 4 promoter^82^.

### Histone acetylation and histone methylation

It has been confirmed that different histone modifications can occur simultaneously or sequentially in a combinatorial manner to regulate the expression of osteogenic genes during osteogenic differentiation^34,83,84^. In this section, we summarize and discuss regulators controlling the expression of osteogenic marker genes by modulating both histone acetylation and histone methylation levels at these gene promoters (Table 3).

**Table 3:** Histone acetylation and methylation centric-regulation in osteogenic genes expression.

Regulator/histone modifer	Histone modification	Target gene	Role	Reference
NaBu	H3K9Ac, H3K9Me3	Runx2	Promoted osteogenic differentiation of ADSCs	^86^
TSA		BMP, ALP	Promoted osteogenic differentiation of nonosteogenic cells	^87^
Wnt/β-catenin	H3K9Ac, H3K14Ac, H4K12Ac, H3K9Me2	PPARγ2	Attenuated osteogenic potential of osteoporotic BMSCs	^90^
HOXA10	H4Ac, H3K4Me3	Runx2, OC, ALP, BSP	Promoted osteogenic differentiation of osteoprogenitor cells	^92^
miR-23a	H3K27Ac,H3K27Me3	OC	Promoted bone formation in mice	^77^

#### H3K9 acetylation and methylation

In a study aimed at improving the osteogenic potential of adipose tissue-derived stem cells, sodium butyrate, the sodium salt of the short-chain fatty acid butyric acid that functions as an inhibitor of histone deacetylases^85^, reportedly increased the osteogenic differentiation capacity of adipose tissue-derived stem cells following stimulation of osteogenic differentiation-specific genes, including *Runx2*, osteopontin, *OC*, and alkaline phosphatase (*ALP*). Investigation of the molecular mechanisms demonstrated that sodium butyrate enhanced Runx2 transcription by increasing the recruitment of transcriptionally permissive histone modification H3K9Ac, and decreasing the recruitment of transcriptionally repressive histone modification H3K9Me3 at the Runx2 promoter^86^. Furthermore, trichostatin-A, another histone deacetylase inhibitor, was shown to promote the trans-differentiation of non-osteogenic cells (3T3-L1 and NIH3T3) into osteoblasts with enhanced expression of BMP2 and ALP by increasing H3K9Ac and decreasing H3K9Me3 levels at the BMP2 and ALP promoters^87^.

Moreover, a study of the underlying molecular mechanism through which BMSC adipogenesis overwhelms osteogenesis during osteoporosis, revealed that inhibition of Wnt/β-catenin signaling increased the enrichment of H3K9Ac, H3K14Ac, and H4K12Ac and decreased the levels of H3K9Me2 on the promoter of peroxisome proliferator-activated receptor-gamma isoform 2, a lipid-activated transcription factor required for adipogenesis^88,89^, thereby elevating its gene expression. This resulted in the inhibition of osteogenic differentiation and promotion of adipogenic differentiation^90^.

In BMP2-mediated osteogenic differentiation, Hoxa10, an important transcription factor that is essential for bone formation^91^, is rapidly induced and functions as an activator of Runx2, OC, ALP, and BSP transcription by binding to these gene promoters and altering chromatin modification, for example, by increasing the levels of histone H4 acetylation and H3K4Me3. This resulted in enhanced osteogenic differentiation in a Runx2-dependent or Runx-independent manner^92^.

#### H3K27 acetylation and methylation

In a study performed to evaluate the epigenetic mechanisms of miR-23a in regulating bone synthesis and homeostasis, knockdown of the miR-23a cluster was shown to enhance osteogenic differentiation with decreased expression of EZH2 and increased expression of Baf45a, a factor responsible for increasing chromatin accessibility. This resulted in the induction of OC by increasing H3K27ac levels and decreasing H3K27me3 levels at the OC gene promoter^77^.

## Conclusion and perspectives

**Fig. 1:**
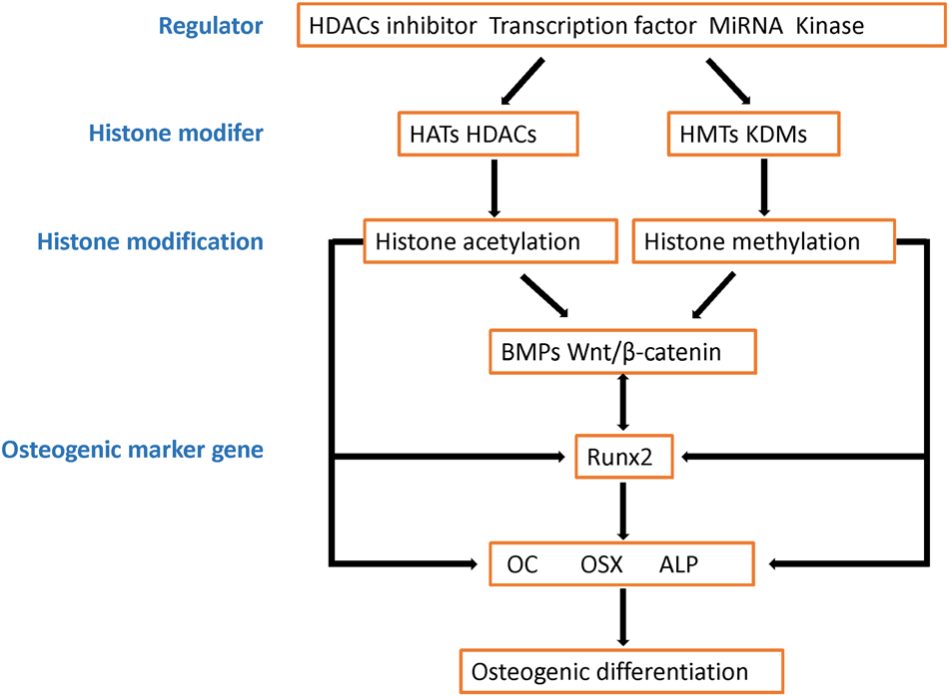
Schematic model of the roles of histone modification in osteogenic marker genes expression. During osteogenic differentiation, numbers of regulators are found to modulate histone acetylation and methylation by affecting the catalytic activity of histone modifiers. The altered histone acetylation and methylation then control expression of osteogenic marker genes, directly or indirectly. HATs histone acetyltransferases, HDACs histone deacetylases, HMTs histone methyltransferases, KDMs histone demethylases, BMPs bone morphogenetic proteins, HOXs homeobox genes, Runx2 runt-related transcription factor 2, OC osteocalcin, OSX osterix, ALP alkaline phosphatase.

To date, several regulators and histone modifiers have been reported to affect osteogenic differentiation by regulating histone modification, thus affecting the expression of osteogenic marker genes. In this review, we summarized and discussed the molecules that control histone modification, as well as the molecular mechanism by which histone modification regulates the expression of its target genes. Most of these molecules are deacetylase inhibitors, transcription factors, miRNAs, and protein kinases, which play important roles in modulating the catalytic activity of histone modifiers, including histone acetyltransferases, histone deacetylases, histone methyltransferases, and histone demethylases. Furthermore, histone acetylation and methylation were found to mediate the BMP and Wnt/β-catenin signaling pathways that are required for osteogenic differentiation, as well as control the expression of Runx2 and Runx2-targeted osteogenic marker genes, including *OC, OSX*, and *ALP* (Fig. 1). Dysregulation of osteogenic differentiation has been linked to several pathophysiologic processes, such as osteopenia, osteopetrosis, osteogenesis imperfecta, and osteoporosis. Thus, histone modifications may be useful therapeutic targets for treating osteogenic differentiation-related diseases after clarifying the main histone modifications and interplay between these histone modifications during disease development. Overall, this review discusses histone modification-centric regulation of osteogenic marker genes and provides insights into its potential clinical utility in osteogenic differentiation-related diseases.
